# Fear of falling as a potential complication of Ramsay Hunt syndrome in older adults: a case report

**DOI:** 10.1186/s12877-022-03606-2

**Published:** 2022-11-24

**Authors:** Chih-Chieh Chou, Yu-Tai Lo, Hui-Chen Su, Chia-Ming Chang

**Affiliations:** 1 Department of Family Medicine, National Cheng Kung University Hospital, College of Medicine, National Cheng Kung University, Tainan, Taiwan; 2 Department of Geriatrics and Gerontology, National Cheng Kung University Hospital, College of Medicine, National Cheng Kung University, No. 138, Sheng Li Road, Tainan City, 70403 Taiwan; 3 Department of Neurology, National Cheng Kung University Hospital, College of Medicine, National Cheng Kung University, Tainan, Taiwan; 4 Department of Medicine, College of Medicine, National Cheng Kung University, Tainan, Taiwan; 5 Institute of Gerontology, College of Medicine, National Cheng Kung University, Tainan, Taiwan

**Keywords:** Ramsay hunt syndrome, Falls, Fear of falling, Older patients, Comprehensive geriatric assessment (CGA)

## Abstract

**Background:**

Fear of falling (FOF) is a common and major health concern in older adults. The consequences of FOF include reduced physical performance, social activity, and health-related quality of life. Ramsay Hunt syndrome (RHS) is a herpes zoster-related facial nerve dysfunction accompanied by an erythematous vesicular rash on the ear or mouth that may complicate ipsilateral facial paralysis and otalgia, vertigo, tinnitus, hearing loss, and meningoencephalitis. However, repeated falls and subsequent FOF due to RHS have not been reported in older adults.

**Case presentation:**

A 65-year-old woman diagnosed with RHS experienced repeated falls during hospital admission and after discharge. Despite recovery of balance and no subsequent falls, the patient presented with persistent FOF at the geriatric outpatient follow-up visit 1 year after the RHS episode. The fear sensation impaired the patient’s instrumental daily activities and was confirmed by documentation of serial comprehensive geriatric assessments, especially the Timed Up and Go test scores.

**Conclusions:**

RHS may cause repeated falls and FOF, leading to impairment in daily activities and psychosocial function in older adults. Therefore, clinicians should be mindful of falls and FOF when caring for older patients with RHS and should develop multidimensional strategies for fall prevention and FOF.

## Background

Falls, defined as “unexpected event [s] in which participants drop to the ground, floor, or lower level” [1], commonly occur in older people, and the incidence of falls increases with age. Approximately 27.5% of adults aged ≥65 years had at least one fall episode within 1 year, and the rate increased to 33.8% for those aged ≥85 years [2]. Falls are the fifth leading cause of death in older adults [3] and increase healthcare utilization and medical costs [4]. Furthermore, falls may develop into fear of falling (FOF). FOF is defined as an exacerbated concern for falling when performing activities of daily living [5]. Depending on different FOF measurement strategies, the prevalence of FOF in older people varies between 21 and 85% [6]. FOF is associated with activity avoidance, loss of self-efficacy, and loss of self-confidence. FOF can lead to restricted social activities and a reduced quality of life [7].

Ramsay Hunt syndrome (RHS), also known as herpes zoster oticus, is caused by reactivation of latent varicella-zoster virus (VZV) in the geniculate ganglion [8]. Ipsilateral facial paralysis, otalgia, and vesicular rash over the external auditory canal comprise the symptom triad of RHS. Abnormal taste, vertigo, tinnitus, and hearing loss have been reported in some patients [9]. After Bell’s palsy, RHS is the second most common cause of non-traumatic peripheral facial paralysis [10]. However, facial paralysis is often more severe in patients with RHS than in those with Bell’s palsy [10]. Additionally, age is the most vital risk factor affecting reactivation of herpes zoster, and approximately 50% of 85-year-old adults suffer from one episode of herpes zoster [11]. Most clinicians focus on facial paralysis and postherpetic neuralgia in patients with RHS. Nevertheless, vertigo caused by RHS may result in repeated falls and fall-related injuries, including FOF, which may profoundly affect patients, especially older adults. To the best of our knowledge, repeated falls and subsequent FOF complicated by RHS have not been reported in older adults. Herein, we report the case of a patient with RHS who initially presented with dizziness and right otalgia followed by repeated falls. In the subsequent period, the patient was bothered by the FOF, which affected the patient’s gait, daily function, and social interactions.

## Case presentation

The patient was a 65-year-old Taiwanese woman with underlying hypertension, diabetes mellitus, dyslipidemia, and chronic hepatitis B virus infection. Her baseline activities of daily living (ADLs) and instrumental activities of daily living (IADLs) indicated complete independence. The patient lived with her husband, and their son lived nearby.

**Fig. 1:**
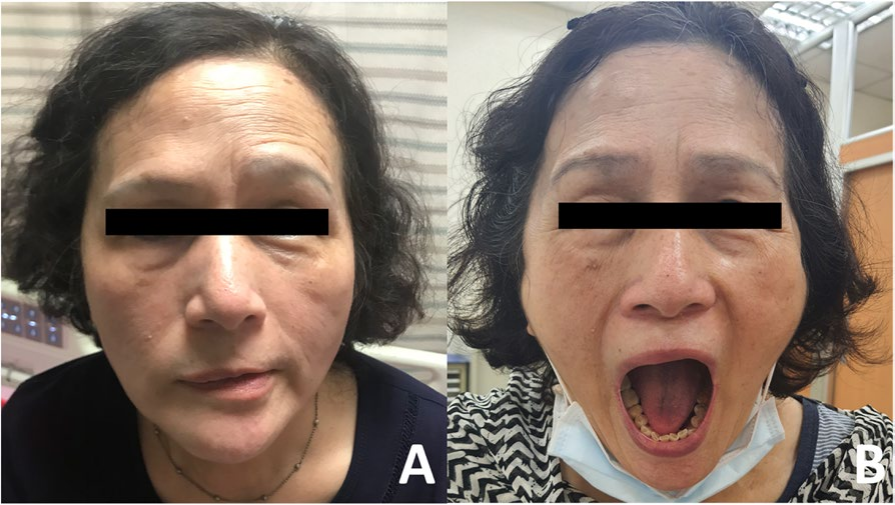
**A** Initial onset of Ramsay Hunt syndrome. Right facial palsy presented as an asymmetric mouth and incomplete closure of the right eye. The patient received a Grade V score on the House–Brackmann Facial Paralysis Scale on December 16, 2019. **B** One year after the patient’s Ramsay Hunt syndrome episode. Her facial palsy was considerably improved, and she received a Grade II score on the House–Brackmann Facial Paralysis Scale on March 30, 2021

**Fig. 2:**
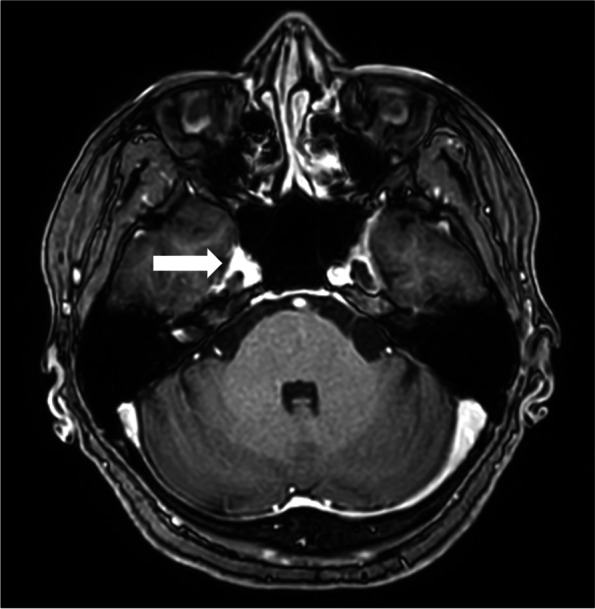
Brain magnetic resonance image. Increased enhancement in the distal right internal auditory canal is evident (arrow)

On presentation, the patient had acute onset of dizziness and right otalgia 15 days before admission. Several vesicles emerged from the patient’s right auricle 1 day later. She was diagnosed with herpes zoster infection in the right ear, and an oral antiviral agent (acyclovir 800 mg QID) was prescribed for 7 days at a local clinic 5 days after initial symptoms. Unfortunately, the dizziness and right otalgia did not improve with treatment. Upon experiencing acute right-sided facial palsy 13 days after initial symptoms, the patient visited the emergency department, where physical examination revealed right peripheral-type facial palsy, presenting as an asymmetric mouth and incomplete closure of the right eye. Oral steroids (prednisolone 5 mg BID) were prescribed for emergent treatment of facial palsy. She was subsequently referred to the neurology clinic. As RHS with 7th and 8th cranial nerves involvement was suspected, the patient was immediately admitted to the neurology department for further evaluation. After admission, the patient’s right peripheral type facial palsy was estimated as Grade V severity on the House–Brackmann Facial Paralysis Scale (Fig. 1A). The Romberg test failed because of the patient’s inability to stand steadily while closing her eyes. The result of the vestibular function test was compatible with right vestibular dysfunction. The otorhinolaryngology fiberoscope displayed two crusts at the right auricle without any other lesions in the right external auditory canal. Brain magnetic resonance imaging (MRI) with gadolinium administration revealed increased enhancement in the distal right internal auditory canal (Fig. 2). A cerebrospinal fluid (CSF) study demonstrated lymphocyte-predominant pleocytosis. Herpes simplex virus and VZV were not identified in the viral nucleic acid polymerase chain reaction of the CSF. No pathogens were detected in the CSF by virus isolation or bacterial culture. Under the suspicion of aseptic meningitis, an empirical intravenous antiviral agent (acyclovir 750 mg Q8H) was administered for 14 days. Artificial tears (hypromellose/dextran 1 gtt QID OU) were prescribed for the patient’s lagophthalmos in the right eye. Physiatrist-arranged electrical stimulation and physical therapy, including self-facial exercises, facial stretches, and mirror visual feedback, were used to treat the patient’s facial palsy. Despite completing the treatment course of intravenous antiviral agents, the patient still experienced dizziness and imbalance and could not ambulate by herself. The patient required assistance or needed to hold a handrail when getting out of bed. After 15 days of hospitalization, the patient was discharged with a stationary clinical manifestation.

After discharge, the patient fell three times at home within 1 month. Due to repeated falls, she visited the geriatric clinic 22 days after discharge and completed a comprehensive geriatric assessment (CGA). The Short Physical Performance Battery (SPPB) revealed poor balance (1/4), poor gait speed (1/4), and fair chair stand (3/4) [12]. The patient completed the Timed Up and Go (TUG) test in 9.4 seconds, which was within the normal range for older adults (Table 1). FOF was confirmed through the positive response to the questions on the FOF and activity restriction (SQ-FAR) questionnaire, which asked, “Are you afraid of falling?” and “If YES, did your FOF lead you to restrict some of your activities?” [14]. The patient was referred to a physical therapy center for rehabilitation and balance training. The physical therapist educated her on home-based self-balance training. The second CGA was conducted 3 months after the first CGA. The patient had had no further falls since the last CGA. However, she still had FOF and refused to go outdoors. The patient’s SPPB revealed resumed balance (4/4) and walking speed (4/4) but deteriorated chair stand (2/4). The patient’s TUG test time increased to 14.1 seconds. At the third CGA performed 3 months after the second CGA, the patient’s TUG test time increased to 16.2 seconds. At the same time, the patient’s IADLs showed new impairments in three aspects: shopping, transportation, and handling of finances. She required family members to accompany her when she left home. The fourth CGA, performed 6 months after the third CGA, showed that the patient’s TUG test time increased to 17.6 seconds, which was higher than in any of her previous TUG tests. The IADLs assessment still showed an impairment in shopping. The patient’s EuroQol-visual analog scales (EQ-VAS) score, which represents a patient’s self-rated health on a vertical visual analog scale, was between 55 to 80 throughout the four CGAs [15]. The patient reported no additional falls between the follow-up appointments, and her handgrip strength was intact throughout the serial CGAs. The patient reported that physical therapy and balance training were useful, and she felt grateful because no incident of falls had occurred afterward. However, even though her SPPB improved, especially during the balance test, the patient’s FOF persisted, and she was too afraid to walk by herself. This consequently caused her to become less socially active due to the fear of going out independently, even though her facial palsy had considerably improved 1 year after the onset of RHS (Fig. 1B).

**Table 1:** Serial Follow-Up of Comprehensive Geriatric Assessments

Time after admission	1st CGA 1 month	2nd CGA 4 months	3rd CGA 7 months	4th CGA 13 months
Body Weight (kg)	68.7	71.2	70.2	70.3
Body Fat (%)	–	36%	35.8%	36.7%
ADLs	12	12	12	12
IADLs	8	8	5	7
CFS	3	3	3	3
MNA-SF	13	14	12	14
SPMSQ	10	10	9	9
GDS-5	1	0	1	1
EQ-5D value^a^	0.694	1	0.694	1
EQ-VAS	70	60	55	80
Falls in the past year	3	–	–	0
SMI^b^ (kg/m^2^)	6.3	6.5	6.5	6.4
Handgrip strength (kg)				
Right	24.1	25.1	19.6	22.4
Left	23.6	22.4	21.3	23.8
Time Up and Go Test (sec)	9.4	14.1	16.2	17.6
SPPB				
Balance Test	1	4	–	4
Gait Speed	1	4	–	3
Chair Stand Test	3	2	–	2
Total	5	10	–	9

*ADLs* activities of daily living, *IADLs* instrumental activities of daily living, *CFS* Clinical Frailty Scale, *MNA-SF* Mini Nutritional Assessment Short Form, *SPMSQ* Short Portable Mental State Questionnaire, *GDS* Geriatric Depression Scale, *EQ-VAS* EuroQol-Visual Analogue Scales, *SMI* Skeletal Muscle Mass Index, *SPPB* Short Physical Performance Battery

^a^Calculated using the Taiwan time trade-off values for EQ-5D health states [13]

^b^SMI was measured by InBody 270 Body Composition Analyzer

## Discussion and conclusions

We report the case of a 65-year-old woman who presented with persistent FOF 1 year after an episode of RHS, even though her serial CGAs showed recovered balance and no additional falls. Previous reports regarding older adults with RHS have mainly focused on the treatment of facial palsy, postherpetic neuralgia, and disease prognosis. However, the impact of RHS on older adults’ physical, psychological, and social functioning has rarely been reported, despite the fact that functional status is one of the most crucial determinants of older adults’ quality of life. To the best of our knowledge, this is the first report to describe a patient with FOF and functional deterioration after an RHS episode.

The classic symptom triad of RHS consists of ipsilateral facial paralysis, otalgia, and vesicular rash over the external auditory canal. Furthermore, VZV could affect not only the 7th cranial nerve but also other cranial nerves, most commonly the 8th cranial nerve. Symptoms of 8th cranial nerve dysfunction, including vertigo, dizziness, poor balance, tinnitus, and hearing loss have been reported in patients with RHS [9]. According to recent studies, 26–37% of patients with RHS developed vertigo [16–18]. One study showed that vertigo in all patients disappeared with conservative treatment during the hospitalization period [18]. Another study reported that vertigo in a patient lasted up to 7 months [16]. Among patients with acute peripheral facial palsy (RHS and Bell’s palsy), older people were more likely to develop dizziness, vertigo, and poor balance than younger ones [18]. In the present study, the 65-years-old patient had the classic RHS triad, as well as dizziness, vertigo, and poor balance. Due to this patient’s age, she may have a higher tendency to develop vertigo.

FOF was previously defined as “post-fall syndrome,” which indicated that it was a psychological trauma after a fall [7]. However, nowadays, we know that FOF is also present among older adults who have no history of falling [19, 20]. Therefore, other factors are likely involved in the development of FOF apart from falls. The etiology of FOF is multifactorial. Age, female sex, low self-rated health status, depression, functional dependence in ADLs, dizziness, and problems with gait and balance were risk factors reported to be associated with FOF [6]. Among them, age remains significantly associated with FOF in various studies. In other words, older adults are particularly prone to FOF. The patient in this study had many associated risk factors, including older age, female sex, previous falls, dizziness, and imbalance. In addition, the patient’s repeated falls were precipitated by dizziness and imbalance. However, after the SPPB improved with no additional falls, her FOF persisted.

According to the literature, FOF can lead to various consequences [6]. Physically, it can cause decreased physical activity or poorer physical health [20–24], and functionally, it can cause avoidance of activities, lack of engagement in any given activity, and eventual loss of functional independence [21, 23, 25–27]. Psychosocially, FOF can lead to reduced social activities [20, 23, 28], depression [29, 30], and low quality of life [20, 21, 28, 29]. Because of FOF, the patient in this study lost her IADLs. In the long term, this further impaired her social activity and led to her social withdrawal. Additionally, over 50% of patients with facial palsy experience psychological distress and social withdrawal [31]. Therefore, it can be surmised that older adults with FOF after RHS are more likely to experience social withdrawal.

Several effective intervention strategies have been proven to manage FOF, including exercise [32], cognitive behavioral therapy [33], Tai Chi combined with cognitive behavioral therapy [34], and guided relaxation with imagery [35]. CAPABLE (Community Aging in Place—Advancing Better Living for Elders), a home-based program enhancing individual capacity and home environmental supports, can help subjects improve their confidence when performing ADLs without falling [36]. Furthermore, understanding the risk factors of FOF may be useful in developing multidimensional strategies to address FOF [6]. The patient in this study was referred to a physical therapy center for balance training, and the patient’s SPPB improved within 3 months.

In the TUG test, the participants are asked to rise from a chair, walk 3 m, turn, walk back to the chair, and sit down. It measures balance, gait speed, and functional capacity [37]. According to a previous study, the fear of falling in community-dwelling older women is associated with frailty, dynamic balance, and gait deficit. Furthermore, the TUG test predicts a fear of falling in this population [38]. In our study, the patient’s SPPB and balance test improved after rehabilitation; however, she needed more time to complete the TUG test. Based on the patient’s minor reduction of IADL and self-reported FOF, we suspected that the increase in TUG score was related to FOF.

This report completed a year-round serial CGA follow-up, including balance, ADLs, IADLs, muscle strength, and fall assessments. We observed functional changes in this patient after RHS. The limitation of this report is the lack of instruments used to quantify FOF, such as the amended Falls Efficacy Scale or Survey of Activities and FOF in the Elderly. However, the SQ-FAR is still a well-recognized screening tool for FOF [14].

In conclusion, RHS not only results in peripheral facial palsy and otalgia but can also cause dizziness and imbalance, which are risk factors for FOF. In older adults, FOF is a complex health concern that can result in impairment of daily activities and psychosocial functioning. A comprehensive understanding of FOF can assist in the early detection and initiation of interventions with which to reduce its negative consequences. Clinicians should be cautious about falls and FOF, especially in patients with RHS, and should develop multidimensional strategies to prevent functional loss and quality of life impairment after RHS.

## Data Availability

The datasets generated and/or analyzed during the current study are not publicly available due to them containing information that could compromise research participant privacy but are available from the corresponding author YTL on reasonable request.

## Abbreviations

FOF: Fear of falling
RHS: Ramsay Hunt syndrome
ADLs: Activities of daily living
IADLs: Instrumental activities of daily living
CSF: Cerebrospinal fluid
MRI: Magnetic resonance imaging
CGA: Comprehensive geriatric assessment
SPPB: Short Physical Performance Battery
TUG: Timed Up and Go
SQ-FAR: Single question on FOF and activity restriction
EQ-VAS: EuroQol-visual analog scales
